# Population-Level Access to Breast Cancer Early Detection and Diagnosis in Nigeria

**DOI:** 10.1200/GO.23.00093

**Published:** 2023-12-14

**Authors:** Adeleye D. Omisore, Elizabeth J. Sutton, Racheal A. Akinola, Anuoluwapo G. Towoju, Adenike Akhigbe, Uzoamaka R. Ebubedike, Gavin Tansley, Olalekan Olasehinde, Amita Goyal, Adedoyin Olabisi Akinde, Olusegun I. Alatise, Victoria Lee Mango, T. Peter Kingham, Gregory C. Knapp

**Affiliations:** ^1^ Department of Radiology, Obafemi Awolowo University, Ile-Ife, Nigeria; ^2^ Department of Radiology, Breast Imaging Service, Memorial Sloan Kettering Cancer Center, New York, NY; ^3^ Department of Radiology, Lagos State University Teaching Hospital, Lagos, Nigeria; ^4^ Department of Radiology, University of Benin Teaching Hospital, Benin, Nigeria; ^5^ Department of Radiology, Nnamdi Azikiwe University, Amawbia, Nigeria; ^6^ Department of Surgery, Division of General Surgery, University of British Columbia, Vancouver, BC, Canada; ^7^ Department of Surgery, Obafemi Awolowo University, Ile-Ife, Nigeria; ^8^ Department of Surgery, Division of General Surgery, Dalhousie University, Halifax, NS, Canada; ^9^ Department of Surgery, Hepatobiliary Service, Memorial Sloan Kettering Cancer Center, New York, NY

## Abstract

**PURPOSE:**

Mammography, breast ultrasound (US), and US-guided breast biopsy are essential services for breast cancer early detection and diagnosis. This study undertook a comprehensive evaluation to determine population-level access to these services for breast cancer early detection and diagnosis in Nigeria using a previously validated geographic information system (GIS) model.

**METHODS:**

A comprehensive list of public and private facilities offering mammography, breast US, and US-guided breast biopsy was compiled using publicly available facility data and a survey administered nationally to Nigerian radiologists. All facilities were geolocated. A cost-distance model using open-source population density (GeoData Institute) and road network data (OpenStreetMap) was used to estimate population-level travel time to the nearest facility for mammography, breast US, and US-guided biopsy using GIS software (ArcMAP).

**RESULTS:**

In total, 1,336 facilities in Nigeria provide breast US, of which 47.8% (639 of 1,336) are public facilities, and 218 provide mammography, of which 45.4% (99 of 218) are public facilities. Of the facilities that provide breast US, only 2.5% (33 of 1,336) also provide US-guided breast biopsy. At the national level, 83.1% have access to either US or mammography and 61.7% have access to US-guided breast biopsy within 120 minutes of a continuous one-way travel. There are differences in access to mammography (64.8% *v* 80.6% with access at 120 minutes) and US-guided breast biopsy (49.0% *v* 77.1% with access at 120 minutes) between the northern and southern Nigeria and between geopolitical zones.

**CONCLUSION:**

To our knowledge, this is the first comprehensive evaluation of breast cancer detection and diagnostic services in Nigeria, which demonstrates geospatial inequalities in access to mammography and US-guided biopsy. Targeted investment is needed to improve access to these essential cancer care services in the northern region and the North East geopolitical zone.

## INTRODUCTION

Breast cancer is an increasingly prevalent condition across sub-Saharan Africa.^1^ In Nigeria, the age-standardized incidence rate of breast cancer may be as high as 63 of 100,000.^1-3^ With an age-standardized mortality rate of 25.5 of 100,000, breast cancer is the leading cause of cancer-related death in Nigeria, followed by cervical cancer (13.2 of 100,000).^4^ Delays in diagnosis play a role in the excess mortality of breast cancer in low- and middle-income countries (LMICs) like Nigeria. Resource-stratified guidelines (eg, Breast Health Global Initiative) recommend that even in a low-resource setting, access to mammography and ultrasound (US) is considered essential for the diagnosis and management of breast cancer.^5^ For the pathologic diagnosis, which requires immunohistochemistry for estrogen and progesterone receptor status, US-guided core needle biopsy is the standard of care in high-income countries (HICs). However, image-guided biopsy is not widely available in LMICs where breast cancer is typically diagnosed by blind biopsy or surgical excision.^6^

**CONTEXT**

**Key Objective**
The objective of this study was to quantify the geospatial access to diagnostic imaging infrastructure essential for the management of breast cancer in Nigeria.
**Knowledge Generated**
Over 80% of Nigerians have access to diagnostic imaging services required for the detection of early-stage breast cancer. Access to breast ultrasound (US) is widespread; however, only 2.5% of facilities offer US-guided breast biopsy and large disparities exist in access between the north and south of the country.
**Relevance**
Targeted investment in training and infrastructure development in northern Nigeria and the North East states in particular could have a significant impact on population-level access to breast cancer diagnostics and outcomes.

In Nigeria, women can access diagnostic imaging (DI) services directly (self-referral) or as ordered by a health care provider from either the public (government institutions) or private (not-for-profit or for-profit) system. Despite coverage for diagnostic mammography through the Nigerian National Health Insurance Scheme, the majority of women pay out of pocket for the service.^7^ There is a dearth of data on population-level access to breast imaging that targeted training or health system investment could potentially address in the Nigerian context. Geographic information system (GIS) methodology allows for visual and spatial analyses of health system data and is a useful tool for designing effective cancer prevention and control programs.^8,9^ International and regional research endeavors have used GIS methodologies to showcase the diverse applications of GIS in breast cancer control, including analyzing access to screening and treatment services, identifying geographic risk factors, and informing targeted interventions for improved breast cancer outcomes.^10-12^ This study used a previously validated GIS model to measure population-level access to mammography, breast US and US-guided breast biopsy in Nigeria.

## METHODS

A comprehensive list of public and private facilities offering mammography and US was compiled using publicly available data from the Nigerian Ministry of Health (MOH) accessed on March 18, 2021.^13^ To identify additional facilities offering mammography and US not captured by the MOH data and to capture facilities with the ability to perform US-guided breast biopsy, an electronic survey was developed and administered to 97 Nigerian radiologists; 57 active members of the Breast Imaging Society of Nigeria (BISON) from May to September 2021, and a convenience sampling of 40 additional radiologists to ensure coverage from each of the six geopolitical zones (North Central, North East, North West, South East, South West, South South) in Nigeria. BISON is the professional society for breast imagers in Nigeria and captures the operational awareness of the country's major breast-focused nongovernmental organizations (NGOs), private sector providers, and academic institutions. The survey elicited cross-sectional data on the DI facilities offering mammography and breast US and additional data on the facilities offering US-guided biopsy, including their location, type of facility (private *v* public), US-guided breast biopsy training, and the volume of US-guided biopsy procedures. Public facilities were defined as those that are government-owned and operated versus private facilities that are owned by individuals, corporations, or NGOs, which may operate as either not-for-profit or for-profit entities. A complete survey, including DI facility location, provider training, and procedure volumes, was obtained from 51 respondents. These data were added to the publicly available data from the MOH. Descriptive statistics were presented in percentages for categorical variables and means and standard deviations (SDs) for continuous variables.

### Geospatial Data

All DI facilities were geolocated using Google Earth (Google, Mountain View, CA). These data were subsequently input into a previously published GIS model for measuring access to cancer care in Nigeria.^14-16^ This model consists of an open-source, geopopulated national road network obtained from OpenStreetMap (OpenStreetMap Foundation, Cambridge, United Kingdom) and a 1,000-m^2^ population-density grid on the basis of the 2006 census adjusted to match the United Nation's national population estimates for 2021 (WorldPop Project). Roads were classified as primary (national highways that connect cities and states), secondary (intrastate roads that connect towns and districts/villages within a state), and tertiary (unpaved roads or minor/side roads that lead to secondary/district roads). Using ArcMAP v10.8.2 (Esri, Redlands, CA), road data were cleaned and topographically verified. Road speed data were based on national traffic laws whereby primary, secondary, and tertiary roads were assigned speed limits of 80, 50, and 30 km/h, respectively.^14^

### Measuring Access

A cost-distance model used in our previously published studies was used to estimate population-level travel time to the nearest DI facility for this study.^14,15^ This method calculates the cumulative time associated with travel from any point in the country to a DI facility using the most time-efficient route over the national road network. Any portion of travel over terrain without roads was assigned a walking speed of 5 km/h. Travel time thresholds of 60, 120, and 240 minutes were used in this study to facilitate comparison between regions. These thresholds are not independently associated with specific outcomes. However, 240 minutes of continuous one-way travel is a conservative estimate of what is possible in a single day during daylight hours (ie, 12 hours roundtrip, including intervention). Access was measured nationally and by region (ie, North *v* South) and geopolitical zone (ie, South West *v* South South *v* South East *v* North West *v* North Central *v* North East).

### Ethics

This study was granted research ethics board approval (ERC/2021/07/17) by Obafemi Awolowo University Teaching Hospitals Complex.

## RESULTS

### DI Study Data

**FIG 1:**
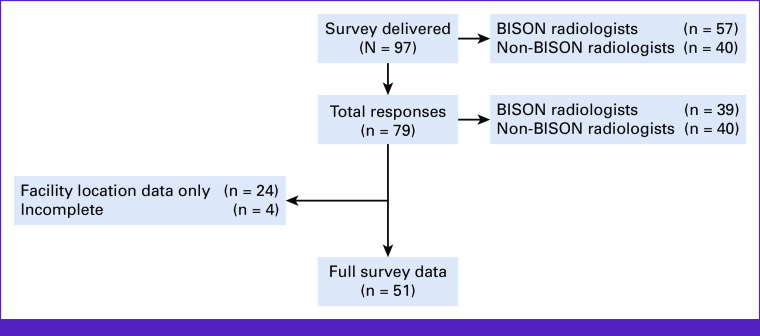
Survey recipients and data capture. BISON, Breast Imaging Society of Nigeria.

In total, 97 Nigerian radiologists were provided with the survey. Among active BISON members, the response rate was 68.4% (39 of 57) versus 100% (40 of 40) from the non–BISON-affiliated radiologists. In total, data on DI facility location were obtained from a total of 79 radiologists, which included 51 complete surveys with responses to all data fields (Fig 1). Respondents provided data for an additional 275 US facilities and 139 mammography facilities not captured by the MOH data set. The survey data were combined with the comprehensive facility data provided by the MOH.

### Breast Imaging Capacity and Training of Nigerian Radiologists

**FIG 2:**
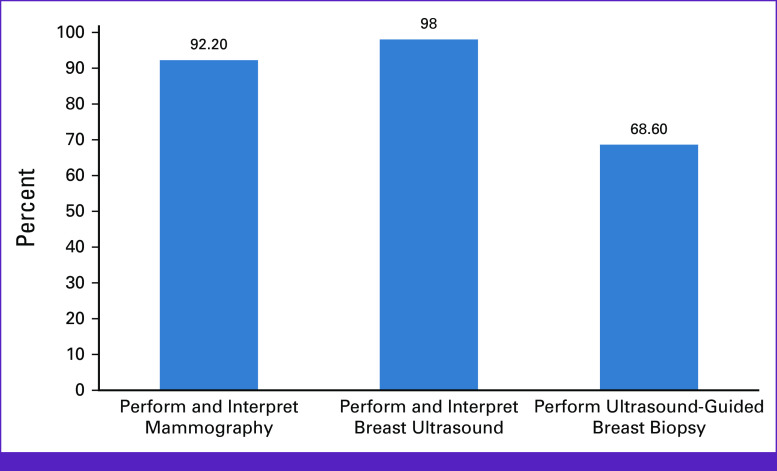
Diagnostic imaging services provided by surveyed Nigerian radiologists.

Overall, 92.2% (47 of 51) of surveyed radiologists interpret mammography, whereas 98.0% (50 of 51) perform and interpret breast US. Of those performing breast US, 68.6% (35 of 51) perform US-guided breast biopsy (Fig 2). Among radiologist performing US-guided breast biopsy, 51.4% (18 of 35) of respondents received training in the procedure during residency or fellowship versus 34.3% (12 of 35) trained/mentored while in practice. Radiologists performing US-guided breast biopsies have been doing so for a mean of 6.5 years (SD, 4.8).

### Distribution of Facilities

**FIG 3:**
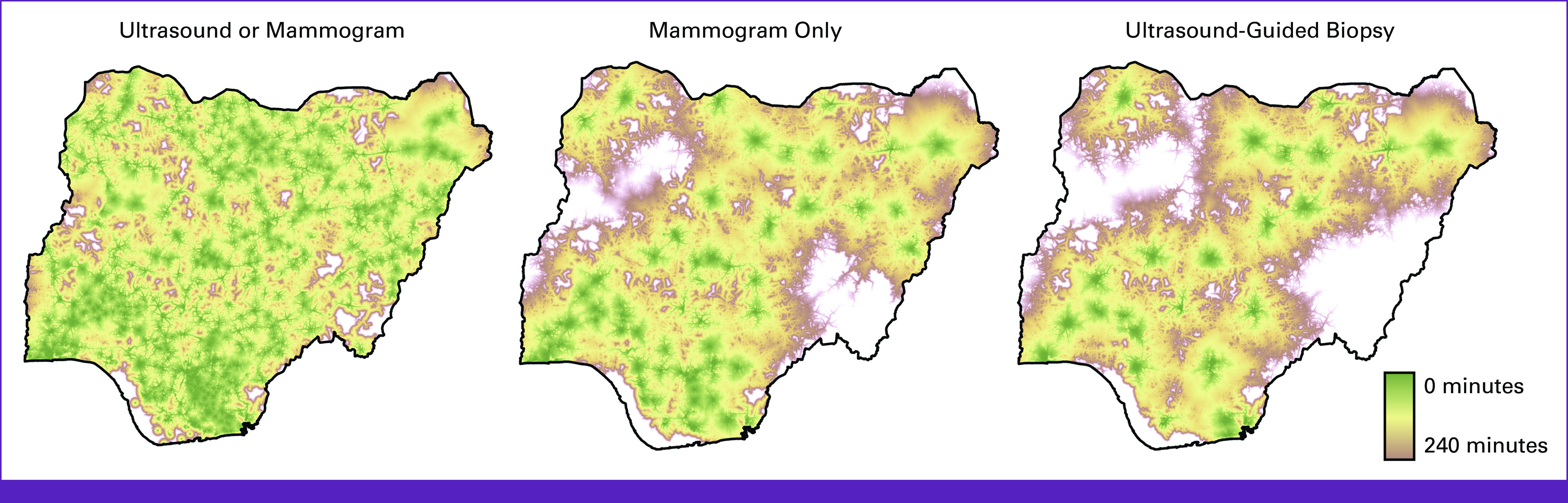
Population-level access to breast imaging and US-guided biopsy in Nigeria. US, ultrasound.

Across the country, 1,336 DI facilities perform breast US, of which 47.8% (639 of 1,336) are publicly administered. US services are available in every state and geopolitical zone of the country. In total, 218 DI facilities offer mammography, of which 45.4% (99 of 218) are publicly administered. Taraba State (North East) and Zamfara State (North West) do not have any mammography capacity. Only one mammography facility was identified in Jigawa State (North West; Fig 3). Of note, all 218 facilities that offer mammography also offer US. Only 2.5% (33 of 1,336) of the US facilities in Nigeria offer US-guided breast biopsy. Seventy-three percent (72.7%, 24 of 33) of facilities with US-guided breast biopsy services are publicly administered.

### Population-Level Access to Early Breast Cancer Detection and Diagnosis

**FIG 4:**
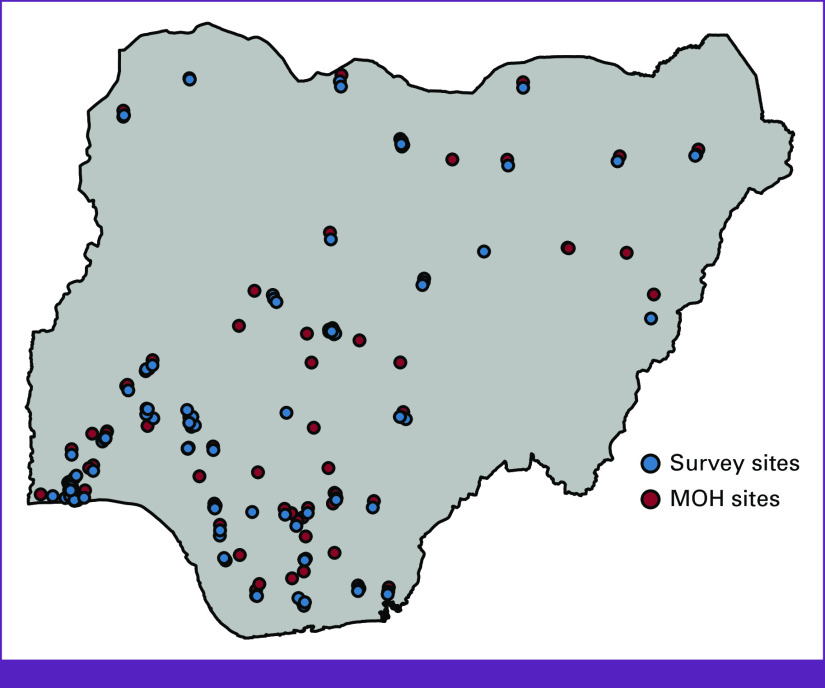
Location of diagnostic imaging centers offering mammography in Nigeria. MOH, Ministry of Health.

Access to DI for early breast cancer detection at the national level is robust in Nigeria, with 83.1% of the population within 120 minutes of travel to a facility with either mammogram or breast US (Fig 4). Across the six geopolitical zones, population-level access to breast US is >80% at 120 minutes of continuous one-way travel, with access between the north (83.7%) and south (82.7%) near parity. For mammography, the national population-level access is 72.1% at 120 minutes (Table 1). However, population-level access to mammography varies by geopolitical zone and region (Fig 4). In the South East, 85.1% of the population has access to mammography within 120 minutes of continuous travel compared with 57.9% in the North East. In southern Nigeria, 80.6% of the population has access at 120 minutes versus just 64.8% in the north.

**TABLE 1:**
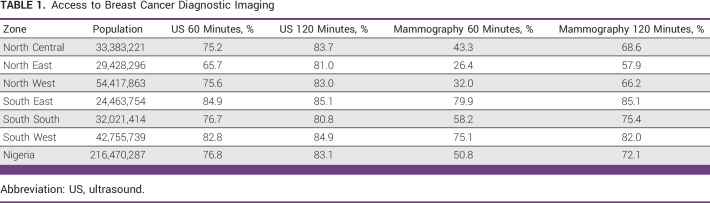
Access to Breast Cancer Diagnostic Imaging

To establish a pathologic diagnosis, a biopsy is required. Nationally, population-level access to US-guided breast biopsy is 61.9% at 120 minutes. This improves to 80.8% when continuous travel is extended to 240 minutes (Table 2). Similar to mammography, access to US-guided breast biopsy varies considerably between the geopolitical zone and the region. Within 60 minutes of continuous travel from the place of residence, 62.6% of the population in the South West geopolitical zone has access to US-guided breast biopsy compared with a mean of just 23.1% in the rest of the country. Although inequalities in access to US-guided breast biopsy improve as travel time increases, parity is not reached for US-guided breast biopsy by 240 minutes of continuous one-way travel. In the North East geopolitical zone, 68.7% of the population has access to US-guided biopsy within 240 minutes of travel versus 85.4% in the South East. Outside of the North East, the remaining five geopolitical zones have >80% population-level access to this essential service within 240 minutes of travel (Table 2).

**TABLE 2:**
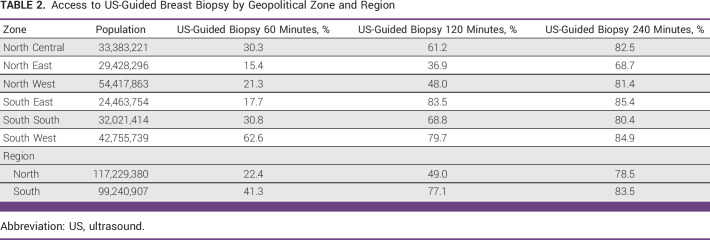
Access to US-Guided Breast Biopsy by Geopolitical Zone and Region

## DISCUSSION

In this study, to our knowledge, a comprehensive assessment of DI facilities essential for the detection, diagnosis, and management of breast cancer in Nigeria is presented for the first time. The relationship between the location of DI facility, including US-guided breast biopsy, and population density reveals important inequalities in access between geopolitical zones and, notably, between northern and southern regions of the country. In the densely populated and relatively urban South West, access to mammogram, US, and US-guided breast biopsy is attainable within a day's roundtrip travel for >80% of the population. Similar to our previous work evaluating access to the country's comprehensive cancer centers, the northern half of the country and the North East geopolitical zone in particular remain outliers in population-level access to essential DI services.^14^ The relatively better access to US-guided breast biopsy in the south can be partially attributed to ongoing National Institute of Health (NIH)–funded capacity building for Nigerian radiologists in US-guided breast biopsy using mobile Health technology (NIH/National Cancer Institute R21 CA239784 grant). The initial phase of this program is domiciled in eight tertiary public hospitals in the South West and South East geopolitical zones.

Increasing the capacity of radiologists to perform US-guided biopsy in northern Nigeria and the North East geopolitical zone will improve access to this essential service and reduce geographic inequalities in access. Security issues in northern Nigeria, particularly in the North East, has negatively affected development in this part of the country including access to health care services.^17,18^ Regional differences in infrastructures, such as road and communication networks, further exacerbate inequalities in population-level access to essential health care services, including cancer care.^19^ Improving basic infrastructure across the north of Nigeria will improve access to breast cancer early diagnosis and detection.

Resource-stratified breast cancer guidelines from the National Comprehensive Cancer Network and the Breast Health Global Initiative include access to mammography and US as the minimum standard of care.^6^ The Nigerian National Cancer Control Plan (2018-2022) identified breast cancer screening and health system strengthening to promote early diagnosis as a priority.^20^ Our study provides baseline data to support this strategic priority. When appropriate in the Nigerian context, breast cancer screening should incorporate the International Agency for Research on Cancer quality indicators and endeavor to address the dearth of West African data in the Cancer Screening on 5 Continents (CanScreen5) project.^21^ A baseline assessment of health resource availability and physical location is required to inform both public and private investment in system expansion. To maximize the impact of additional investment, the underserved region (northern Nigeria) and states (Taraba, Zamfara and Jigawa) that have severely limited access to mammography should be prioritized.

Timely access to DI services has a direct impact on cancer diagnosis and screening uptake and retention. For colorectal cancer, travel time has been associated with participation in screening and diagnosis, with an odds ratio (OR) of 0.86 (95% CI, 0.77 to 0.93) for each 10-minute increase in travel time from the center offering colonoscopy for patients with a positive fecal immunochemistry test.^22^ Specific to breast cancer, numerous studies, mostly from HICs, demonstrate a cost-distance relationship where physical travel time is inversely related to uptake, screening retention, and outcomes.^23^ In the United States, women have to live within 10 km of a DI facility with mammography to see a significant increase in uptake (OR, 1.26 [95% CI, 1.09 to 1.47]).^24^ Our previous work in Nigeria demonstrated a significant increase in the odds of presenting with advanced-stage disease among patients in the highest travel time quintile compared with the lowest quintile (OR, 2.82 [95% CI, 1.30 to 6.11]).^15^ In our current study, population-level access to mammography within 60 minutes was just 50.9%. As a high-income benchmark, >85% of women have access to mammography within 20 minutes in the Unites States.^25^ At Obafemi Awolowo University Teaching Hospital, Ile-Ife, the travel time of >30 minutes was associated with significantly worse outcomes (OR of death 1.65 [95% CI, 1.17 to 2.33]) compared with those who live <30 minutes from the diagnostic/treatment center.^15^ In a recent retrospective analysis at two tertiary care facilities in Nigeria, only 44% of patients received a mammography and 41% received an US as a component of breast cancer diagnosis and management.^26^ The low rates of mammography and US use in Nigeria play a role in the consistently poor disease-free and overall survival rates among Nigerian patients with breast cancer.^27^ Inequalities in access will disproportionately affect patients of lower-socioeconomic status as the fixed, out-of-pockets costs associated with travel are inherently regressive.

There are several important limitations to consider when examining the results of this study. The DI data represent a cross-sectional analysis that is in continuous evolution, in terms of both physical location and functionality. To the best of our knowledge, every center included was operational at the time of analysis. However, service disruption because of power outages, human resource constraints, and maintenance are common. To compile a comprehensive list of available facilities, we used updated data from the Nigerian MOH, a survey administered to the representative national professional association (ie, BISON) and region-specific stakeholders. The end result is the most complete data set currently available for mammography, US, and US-guided breast biopsy in Nigeria. We hypothesize that the discrepancy between the MOH and survey data may reflect the rapid expanse of DI facilities across the country in the past 10 years. However, data may still be missing from this analysis. Data on procedure volume and training were collected via voluntary survey and would be subject to selection and recall bias. Finally, because of the availability of reliable sex-based population-level data, our analysis was not limited to women or women potentially susceptible to breast cancer on the basis of age (eg, ≥18-year-old); however, this should not affect the interpretation of our results as gender distribution is expected to be fairly uniform across the country.

In conclusion, the rising incidence of breast cancer and breast cancer–related mortality in Nigeria requires a comprehensive approach to cancer system planning and delivery. A fundamental component of improving access is understanding the physical distribution of available resources for breast cancer early diagnosis/detection in relation to the population. At present, >80% of the Nigerian population has timely access to either mammography or US. However, large geographic variability exists between the north and south and between geopolitical zones. Inequalities in access will exacerbate differences in breast cancer–specific outcomes unless addressed by targeted investment and data-driven cancer system development. The results from this study will inform strategies for implementation for breast cancer early diagnosis/detection.
